# Antimicrobial Activity of Five Herbal Extracts Against Multi Drug Resistant (MDR) Strains of Bacteria and Fungus of Clinical Origin

**DOI:** 10.3390/molecules14020586

**Published:** 2009-02-04

**Authors:** Rosina Khan, Barira Islam, Mohd Akram, Shazi Shakil, Anis Ahmad, S. Manazir Ali, Mashiatullah Siddiqui, Asad U. Khan

**Affiliations:** 1 Interdisciplinary Biotechnology Unit, Aligarh Muslim University, Aligarh 202002, India; E-mails: ruzinakhan3@hotmail.com (R.K.), barira.khan@gmail.com (B. I.), akramwali@yahoo.co.in (M. A.), shazicool@rediffmail.com (S. S.), asadukhan72@gmail.com (A-U. K.); 2 Department of Pediatrics, J N Medical College and Hospital, AMU, Aligarh, India; E-mail: manazir1958@yahoo.com (S-M. A.); 3 Department of Biochemistry, J N Medical College and Hospital, AMU, Aligarh, India; E-mail: Mashiatsiddiqi81@gmail.com (M. S.)

**Keywords:** Herbal extracts, Antimicrobial agent, Multi-drug resistant.

## Abstract

Antimicrobial activities of the crude ethanolic extracts of five plants were screened against multidrug resistant (MDR) strains of *Escherichia coli, Klebsiella pneumoniae* and *Candida albicans.* ATCC strains of *Streptococcus mutans, Staphylococcus aureus, Enterococcus faecalis, Streptococcus bovis, Pseudimonas aeruginosa, Salmonella typhimurium, Escherichia coli, Klebsiella pneumoniae* and *Candida albicans* were also tested. The strains that showed resistance against the maximum number of antibiotics tested were selected for an antibacterial assay. The MDR strains were sensitive to the antimicrobial activity of *Acacia nilotica*, *Syzygium aromaticum* and *Cinnamum zeylanicum*, whereas they exhibited strong resistance to the extracts of *Terminalia arjuna* and *Eucalyptus globulus*. Community-acquired infections showed higher sensitivity than the nosocomial infections against these extracts. The most potent antimicrobial plant was *A. nilotica* (MIC range 9.75-313µg/ml), whereas other crude plant extracts studied in this report were found to exhibit higher MIC values than *A. nilotica* against community acquired as well as nosocomial infection. This study concludes that *A. nilotica*, *C. zeylanicum* and *S. aromaticum* can be used against multidrug resistant microbes causing nosocomial and community acquired infections.

## Introduction

Antibiotics provide the main basis for the therapy of microbial (bacterial and fungal) infections. Since the discovery of these antibiotics and their uses as chemotherapeutic agents there was a belief in the medical fraternity that this would lead to the eventual eradication of infectious diseases. However, overuse of antibiotics has become the major factor for the emergence and dissemination of multi-drug resistant strains of several groups of microorganisms [1]. The worldwide emergence of *Escherichia coli, Klebsiella pneumoniae, Haemophilus* and many other ß-lactamase producers has become a major therapeutic problem. Multi-drug resistant strains of *E. coli* and *K. pneumoniae* are widely distributed in hospitals and are increasingly being isolated from community acquired infections [2, 3]. *Candida albicans*, also a nosocomial pathogen, has been reported to account for 50-70% cases of invasive candidiasis [4]. Alarmingly, the incidence of nosocomial candidemia has risen sharply in the last decade [5]. All this has resulted in severe consequences including increased cost of medicines and mortality of patients.

Thus, in light of the evidence of rapid global spread of resistant clinical isolates, the need to find new antimicrobial agents is of paramount importance. However, the past record of rapid, widespread emergence of resistance to newly introduced antimicrobial agents indicates that even new families of antimicrobial agents will have a short life expectancy [6]. For this reason, researchers are increasingly turning their attention to herbal products, looking for new leads to develop better drugs against MDR microbe strains [7].

For thousands of years, natural products have been used in traditional medicine all over the world and predate the introduction of antibiotics and other modern drugs. The antimicrobial efficacy attributed to some plants in treating diseases has been beyond belief. It is estimated that local communities have used about 10% of all flowering plants on Earth to treat various infections, although only 1% have gained recognition by modern scientists [8]. Owing to their popular use as remedies for many infectious diseases, searches for plants containing antimicrobial substances are frequent [9]. Plants are rich in a wide variety of secondary metabolites such as tannins, alkaloids and flavonoids, which have been found *in vitro* to have antimicrobial properties [10]. A number of phytotherapy manuals have mentioned various medicinal plants for treating infectious diseases due to their availability, fewer side effects and reduced toxicity [11]. There are several reports on the antimicrobial activity of different herbal extracts [12,13,14]. Many plants have been found to cure urinary tract infections, gastrointestinal disorders, respiratory diseases and cutaneous infections [15, 16]. Cytotoxic compounds have been isolated from the species of *Vismia* [17]. Antibacterial activity of the essential oil as well as eugenol purified from *Ocimum gratissimum* to treat pneumonia, diarrhea and conjunctivitis has also been reported earlier [18]. According to the WHO, medicinal plants would be the best source for obtaining variety of drugs [19]. These evidences contribute to support and quantify the importance of screening natural products. The aim of the present study was to investigate the antibacterial and antifungal activity of ethanolic extracts of *Acacia nilotica, Terminalia arjuna, Eucalyptus globulus, Syzygium aromaticum* and *cinnamomum zeylanicum* against multi-drug resistant strains isolated from nosocomial and community acquired infections.

## Results and Discussion

In this study, we have tested the ethanolic extracts of five plants for their antimicrobial activity against multi-drug resistant strains. ATCC strains of Gram-negative bacteria, Gram-positive bacteria and yeast species were also used as control sensitive strains. All the plant extracts showed antimicrobial activity against at least four of the types of microorganisms tested, as exhibited by an agar diffusion assay (Table 1). Extracts of *A. nilotica, C. zeylanicum* and *S. aromaticum* showed the most potent activity against all the microorganisms studied. *E. faecalis, S. aureus, S. bovis* and *S. mutans* were the most susceptible to all the plant extracts tested. On the contrary, *S. typhimurium, K. pneumoniae, E. coli, P. aeruginosa* and *C. albicans* strains were found to be sensitive to extracts of *A. nilotica, C. zeylanicum* and *S. aromaticum*.

**Table 1:** Susceptibility pattern of crude ethanolic herbal extracts against different microorganisms.

Microbial Strains	Susceptibility pattern of crude herbal extract against different microorganisms^#^				
	A.nilotica^*^	T.arjuna^*^	E.globulus^*^	S.aromaticum^*^	C.zeylanicum^*^
*S. mutans*					
ATCC-700610	+++	+++	++	+++	+++
*S.aureus*					
ATCC-29213	+++	+++	++	+++	+++
*E.faecalis*					
ATCC-29212	++	++	+	++	+++
*S.bovis*					
ATCC 9809	++	++	+	+++	+++
*P.aeruginosa*					
ATCC-27853	+++	-	-	+++	+++
*S. typhimurium*					
ATCC-13311	++++	-	-	+++	+++
*E.coli*					
ATCC-25922	+++	-	-	++	++
*C.albicans*					
ATCC-10231	+++	-	-	++++	++++
*K.pneumoniae*					
ATCC-700603	++	-	-	+	++
*E.coli* [10]^a)^	++ (10/10)	- (10/10)	- (10/10)	+ (10/10)	++ (10/10)
*E.coli* [16]^b)^	- (1/16)			- (1/16)	- (1/16)
	+ (1/16)	- (16/16)	- (16/16)	+ (5/16)	+ (13/16)
	++ (14/16)			++ (10/16)	++ (2/16)
*C.albicans* [18]^c)^				++ (3/18)	
	++ (18/18)	- (18/18)	- (18/18)	+++ (12/18)	++++ (18/18)
				++++ (3/18)	
*K. pneumoniae* [14]^d)^					
	+ (9/14)	- (14/14)	- (14/14)	+ (12/14)	++ (14/14)
	++ (5/14)			++ (2/14)	

^#^ Diameter of inhibition zone: no inhibition (-); 5-15 mm (+); 16-25 mm (+ +); 26-35 mm (+ + +)

> 40 mm (+ + + +)

* values in parentheses indicate number of isolates out of total isolates tested

^a)^ ^& c)^ = isolates of nosocomial infection; ^b)^^&^^d)^ = isolates of community acquired infection

Our data revealed that standard ATCC strains of Gram-positive bacteria were more sensitive than Gram-negative ones towards the plant extracts studied. This data is also supported by previous workers [20]. It has been proposed that the mechanism of the antimicrobial effects involves the inhibition of various cellular processes, followed by an increase in plasma membrane permeability and finally ion leakage from the cells [21]. Amongst the tested Gram-negative bacteria, *K. pneumoniae* was found to be the most sensitive, while *S. typhimurium* was the most resistant bacteria. In case of Gram-positive bacteria, *E. faecalis* was the most sensitive, while *S. aureus* was the most resistant strain. *C. albicans* was found to be highly sensitive to the action of *A. nilotica* (least MIC 4.9 µg/mL) followed by *C. zeylanicum* and *S. aromaticum* with the least MIC being 19.5 µg/mL and 156 µg/mL, respectively (Table 2). On the contrary, *C. albicans* was completely resistant against *T. arjuna* and *E. globulus* at the concentrations tested.

In contrast to the previous findings that Gram-negative bacteria are hardly susceptible to the plant extracts in doses less than 2 x 10^5^ µg/mL [22], our results showed inhibition at concentrations as low as 9.75 µg/mL (*A. nilotica*). The variation of susceptibility of the tested microorganisms could be attributed to their intrinsic properties that are related to the permeability of their cell surface to the extracts. Due to the emergence of antibiotic resistant pathogens in hospitals and homes, plants are being looked upon as an excellent alternate to combat the further spread of multidrug resistant microorganisms. In this study, amongst the five plants, the crude extracts of *A. nilotica, C. zeylanicum* and *S. aromaticum* showed good antimicrobial activity against multidrug resistant strains of *K. pneumoniae, E. coli* and *C. albicans* isolated from nosocomial and community acquired infections (Table 3). Extracts of *A. nilotica* was found to be the most active extract against the nosocomial as well as community acquired isolates. The MIC value of the extract of *A. nilotica* against different isolates was found to be in the range of 4.9-313 µg/mL.

**Table 2:** MIC and MBC/MFC values for crude extracts of plant parts against Multi-Drug Resistant strains of Nosocomial and Community Acquired Infections and susceptible standard strains

Microorganism	MIC(µg/mL) and MBC/MFC (µg/mL) of crude herbal extracts																		
	A. nilotica*			*T. arjuna*				*E. globulus*				*S. aromaticum*				*C. zeylanicum*			
	MIC	MBC/MFC		MIC		MBC/MFC		MIC		MBC/MFC		MIC		MBC/MFC		MIC		MBC/MFC	
*S. mutans*	78	313		1560		3130		3130		6250		390		780		195		390	
ATCC-700610																			
*S. aureus*	39	78		780		1560		6250		12500		780		1560		390		1560	
ATCC-29213																			
*E. faecalis*	9.75	78		1560		3130		3130		12500		195		1560		97.5		1560	
ATCC-29212																			
*S. bovis*	39	78		1560		3130		3130		12500		780		1560		195		390	
ATCC-9809																			
*P. aeruginosa*	39	39		-		-		-		-		390		1560		390		1560	
ATCC-27853																			
*S. typhimurium*	9.75	39		-		-		-		-		1560		1560		1560		3130	
ATCC-13311																			
*E. coli*	19.5	39		-		-		-		-		780		1560		390		1560	
ATCC-25922																			
*C. albicans*	4.9	19.5		-		-		-		-		156		156		19.5		78	
ATCC-10231																			
*K. pneumoniae*	9.75	78		-		-		-		-		390		6250		195		3130	
ATCC-700803																			
*E. coli* [10] ^a)^	156	313 (3/10)		-		-		-		-		6250		12500 (10/10)		3130		6250 (7/10) 12500 (3/10)	
	313	625 (7/10)		-		-		-		-						6250			
*E. coli* [16] ^b)^	19.5	39 (3/16)		-		-		-		-		390		1560 (2/16) 3130 (14/16)		195		1560 (11/16) 3130 (5/16)	
	39	156 (13/16)		-		-		-		-		1560				780			
*C. albicans* [18]^c)^	9.5	39 (9/18)		-		-		-		-		390		780 (3/18) 3130 (15/18)		780		1560 (18/18)	
	39	78 (9/18)		-		-		-		-		780							
*K. pneumoniae* [14]^d)^	156	313 (11/14)		-		-		-		-		780		1560 (4/14)		390		1560 (11/14)	
	313	1250 (3/14)		-		-		-		-		1560		3130 (10/14)		780		3130 (3/14)	

MIC = minimum inhibitory concentration; MBC = minimum bactericidal concentration; MFC = minimum fungicidal concentration; a) & c) = isolates of nosocomial infection; b) & d) = isolates of community acquired infection; * value in parentheses indicates number of isolates out of total isolates tested.; – = No activity at the concentration of the extracts tested.

**Table 3:** Resistance Profile of Multi-Drug Resistant Isolates of Nosocomial and Community Acquired Infections.

Microorganism ^a)^	Source of Infection	Resistance Pattern of Antibacterial/Antifungal Agent	Isolates ^b)^
*E. coli* (10)	Nosocomial	Ch,Ci,Cpm,Ac,Ao,Pc,G,Tb,Na,Cf,T	8E,9E,10E
		Ch,Ca,Ci,Cpm,Ac,Ao,Pc,G,Na,Cf,T	2E,7E
		Ch,Ca,Ci,Cpm,Ac,Ao,Pc,G,Tb,Na,Cf,T	3E,6E
		Ch,Ca,Ci,Cpm,Ac,Ao,Pc,G,Na,Cf,T,C	1E
		Ch,Ca,Ci,Cpm,Ac,Ao,Pc,G,Tb,Na,Cf,T,C	4E,5E
*E. coli* (16)	Community Acquired	Ch,Ci,Cpm,Ac,Pc,Na,Nf,C	128E
		Ch,Ca,Cpm,Ac,Ao,Pc,Ak,Tb,Cf	68E
		Ch,Cpm,Ac,Pc,Tb,Na,Nf,T,C	92E, 112E
		Ch,Ca,Ci,Cpm,Ao,Pc,Na,Cf,T	137E
		Ch,Ci,Cpm,Ac,Pc,Ak,Tb,Na,Cf,Nf,T	186E
		Ch,Ca,Cpm,Ac,Ao,Pc,Ak,Na,Cf,Nf,T,C	61E
		Ch,Ca,Ac,Pc,Ak,G,Tb,Na,Cf,Nf,T,C	93E
		Ch,Ca,Cpm,Ac,Ao,Pc,G,Tb,Na,Cf,Nf,T,C	67E, 144E
		Ch,Ca,Ci,Cpm,Ac,Ao,Pc,Ak,G,Tb,Na,Cf,T	158E
		Ch,Ca,Ci,Cpm,Ac,Ao,Pc,Ak,G,Tb,Na,Cf,Nf,T	103E, 133E
		Ch,Ca,Ci,Cpm,Ac,Ao,Pc,Ak,G,Tb,Na,Cf,Nf,T,C	59E,90E,152E
*K. Pneumoniae (14*)	Community Acquired	Ch,Cpm,Ac,Pc,Ak,Tb,Cf,T	173K
		Ch,Ci,Cpm,Ac,Pc,Ak,G,Cf,T	63K
		Ch,Cpm,Ac,Ak,G,Tb,Cf,Nf,T	66K, 155K
		Ch,Ca,Ci,Cpm,Ac,Ao,Pc,G,Tb,T	111K, 141K
		Ch,Cpm,Ac,Pc,Ak,G,Tb,Na,Cf,Nf,T,C	153K
		Ch,Ca,Ci,Cpm,Ao,Pc,G,Tb,Na,Cf,Nf,T	159K
		Ch,Ca,Ci,Cpm,Ac,Ao,Pc,Ak,G,Tb,Na,Cf,Nf	150K
		Ch,Ca,Ci,Cpm,Ac,Ao,Pc,G,Tb,Na,Cf,Nf,T	164K,174K, 192K
		Ch,Ca,Ci,Cpm,Ac,Ao,Pc,Ak,G,Tb,Na,T,C	165K, 194K
		Ch,Ca,Ci,Cpm,Ac,Ao,Pc,Ak,G,Tb,Na,Cf,Nf,T,C	
*C. albicans* (18)	Nosocomial	It,Ns,Ap	2C,10C,15C
		It,Fu,Ap	17C
		It,Ns,Fu,Ap	14C
		It,Ns,Cc,Ap	11C
		It,Kt,Cc,Ap	5C,13C
		It,Kt,Ns,Cc,Ap	12C
		It,Kt,Ns,Fu,Ap	9C,16C
		It,Ns,Cc,Fu,Ap	3C,4C
		It,Kt,Ns,Cc,Fu,Ap	1C,6C,7C,8C,18C

^a)^ = No. of isolates tested in parentheses; ^b)^ = Name of the strains studied in our lab. Antibacterial Agent: Cephalosporins: Ch=Cephalothin (30 µg), Ca=Ceftazideme (30 µg), Ci=Ceftriaxone (30 µg), Cpm=Cefepime (30 µg). Other ß-lactam: Ac=Amoxyclav (30 µg), Ao=Aztreonam (30 µg), Pc=Piperacillin (100 µg). Aminoglycosides:Ak=Amikacin (30 µg), G= gentamycin (10 µg), Tb=Tobramycin (10 µg); Fluoroquinones:Na=Nalidixic acid (30 µg ), Cf=Ciprofloxacin (5 µg). Others: Nf=Nitrofurantoin (300 µg), T=Tetracycline (30 µg), C=Chloramphenicol (30 µg); Antifungal Agents: It=Itraconazole (10 µg), Kt=Ketoconazole (10 µg), Ns=Nystatin (100 units), Cc=Clotrimazole (10 µg), Fu=Fluconazole (10 µg), Ap=Amphotericin (100 units)

Our data show that strains isolated from nosocomial infection were more resistant to the extracts than community acquired infection ones. It was also reported earlier that the resistance to antibiotics as well as mortality is almost two times higher in case of nosocomial infections than in community-acquired infections [23].

*Acacia nilotica* was found to give the most potent antimicrobial extract (Table 2). It is reported to have antimicrobial, antihyperglycemic and antiplasmodial properties [24,25,26]. *Cinnamum zeylanicum* showed next highest activity, followed by *Syzygium aromaticum*. These two plants are known to possess antipyretic activity [27, 28] and essential oils from these two species have been shown to possess antibacterial activities [29]. Eugenol, a compound found in *S. aromaticum*, is reported to have strong antifungal [30] and anti-inflammatory activities [31], and has been investigated for its potential anticarcinogenic effect [32]. The essential oil from *C. zeylanicum* shows antioxidant [33], antibacterial and antifungal activities [34]. *Terminalia arjuna*, a well known herbal cardiac tonic, is also known to possess antimicrobial activity [35, 36]. *Eucalyptus globules*, traditionally used to treat diabetes [37], showed antimicrobial effects only on Gram-positive bacteria (Table 2). *T. arjuna* contains ellagic acid, ethyl gallate, gallic acid and luteolin that exhibits antimutagenic property [38, 39]. It also possesses a significant antioxidant effect, comparable with vitamin E [40]. Plants of the genus *Eucalyptus* have been shown to produce a number of phloroglucinol sesquiterpene- or monoterpene-coupled compounds, namely, the macrocarpals and euglobals. Their biological activities such as HIV-RTase inhibition, granulation inhibition and antiviral effects have been reported [41, 42]. Globulol isolated from the fruit of this plant has been shown to be the major source of its antimicrobial activity [43].

The antimicrobial potency of plants is believed to be due to tannins, saponins, phenolic compounds, essential oils and flavonoids [44]. It is interesting to note that even crude extracts of these plants showed good activity against multidrug resistant strains where modern antibiotic therapy has failed. As per our results, the MIC values for most of the extracts were lower than their MBC/MFC values, suggesting that these extracts inhibited growth of the test microorganisms while being bactericidal/ fungicidal at higher concentrations.

## Conclusions

The ethanolic extracts of *A. nilotica, C. zeylanicum* and *S. aromaticum* could be a possible source to obtain new and effective herbal medicines to treat infections caused by multi-drug resistant strains of microorganisms from community as well as hospital settings. However, it is necessary to determine the toxicity of the active constituents, their side effects and pharmaco-kinetic properties.

## Experimental

### Plant material

Leaves of *A. nilotica*, *E. globulus* and bark of *T. arjuna* were collected from the gardens of AMU, Aligarh, India. *C. zeylanicum and S. aromaticum* were collected from local market of Aligarh. The taxonomic identity of these plants was confirmed at Department of Botany, AMU, Aligarh, India.

### Preparation of plant extracts

Dried leaves of *A. nilotica, E. globulus*, dried bark *T. arjuna*, *C. zeylanicum* and dry buds of *S. aromaticum* were pulverized or grounded to coarse powder, then suspended in 50% or 90% ethanol for 1 or 7 days. After filtration and evaporation of ethanol, the extracts were oven dried at 60^o^C. For experiments, each extract was redissolved in ethanol to the desired concentration.

### Microbial test strains

Clinical strains of *E. coli, K. pneumoniae* and *C. albicans* from nosocomial and community acquired infections were isolated, identified and characterized by conventional biochemical methods [45, 46]. The study includes ESBL producing strains of *E.coli* and *K. pneumoniae* from community acquired infections [3].Other microbial strains investigated were *S. mutans* ATCC-700610, *S. aureus* ATCC-29213, *E. faecalis* ATCC-29212, *S. bovis* ATCC-9809, *P. aeruginosa* ATCC-27853, *S. typhimurium* ATCC-13311, *E. coli* ATCC-25922, *K. pneumoniae* ATCC-700603 and *C. albicans* ATCC- 10231. *S. mutans* were grown in Brain Heart Infusion (BHI) Broth (Himedia Labs, Mumbai, India), rest of the bacteria were grown in Nutrient Broth (Himedia Labs, Mumbai, India) at 37^o^C. The yeast, *C. albicans* were grown in Yeast Peptone Dextrose (YPD) Broth (Himedia Labs, Mumbai, India) at 30^o^C.

### Determination of the strains sensitivity to antibiotics

The susceptibilities of the microbial strains to different antibiotics were tested using disc diffusion method [45, 46]. Antibacterial agents from different classes of antibiotics were used which included cephalothin, ceftazideme, ceftriaxone, cefepime, amoxyclav, aztreonam, piperacillin, amikacin, gentamycin, tobramycin, fluoroquinones, nalidixic acid, ciprofloxacin, nitrofurantoin, tetracycline and chloramphenicol (Himedia Labs, Mumbai, India). For fungal strains the antibiotics used were itraconazole, ketoconazole, nystatin, clotrimazole, fluconazole, amphotericin (Himedia Labs, Mumbai, India).

### Agar diffusion assay

The extracts were tested for antimicrobial activity using agar diffusion on solid media. Soyabean Casein Digest Agar (TS) was used for *S. mutans*, Nutrient Agar for rest of the bacterial strains and YPD Agar for *C. albicans*. The solid agar was punched with 7mm diameter wells. The inoculums (1.5 x 10^8^ CFU/ml) were spread on to their respective agar plants using sterile swabs and then filled with 100µl extracts. The concentrations of the extracts employed were 0.01 g/ml for *A. nilotica* and 0.1g/ml for rest of the extracts. The plates were then incubated at 37^0^C for 24h. After incubation, zone of growth inhibition for each extract was measured.

### Determination of Minimum Inhibitory Concentration and Minimum Bactericidal/Fungicidal Concentration

Strains with inhibition zones were considered sensitive to the extract, those without such a zone were considered resistant. For MIC, two-fold serial dilutions of the extracts were performed. Each inoculum was prepared in its respective medium and density was adjusted to 0.5 Mcfarland standard (10^8^ CFU/mL) and diluted to 1:100 for the broth microdilution procedure. Microtiter plates were incubated at 37^o^C and the MIC was recorded after 24 h. The MIC is the lowest concentration of the compound at which the microorganism tested does not demonstrate visible growth. MBC/MFC were determined by sub-culturing the test dilutions on to a fresh solid medium and incubated further for 18-24 h. The highest dilution that yielded no bacterial/fungal growth on solid medium was taken as MBC/MFC [47].
